# Re-screening adherence to multi-target stool DNA test for colorectal cancer: real-world study in a large national population

**DOI:** 10.1007/s00384-025-04837-6

**Published:** 2025-02-24

**Authors:** Mallik Greene, Timo Pew, Michael Dore, Derek W. Ebner, A. Burak Ozbay, William K. Johnson, John B. Kisiel, A. Mark Fendrick, Paul Limburg

**Affiliations:** 1 Exact Sciences Corporation, Madison, WI USA; 2 Department of Medicine, Duke University, Durham, NC USA; 3 Division of Gastroenterology and Hepatology, Department of Internal Medicine, Mayo Clinic, Rochester, MN USA; 4 Division of General Medicine, Department of Internal Medicine, University of Michigan, Ann Arbor, MI USA

**Keywords:** Adherence, Cologuard, Colorectal cancer screening, Colorectal neoplasms, Mt-sDNA, Re-screening

## Abstract

**Purpose:**

Adherence to colorectal cancer (CRC) re-screening is essential to maximize screening effectiveness. This study assessed adherence to a multi-target stool DNA (mt-sDNA) test among previous users in the USA across different payer types.

**Methods:**

Data from Exact Sciences Laboratories LLC (01/01/2023–12/31/2023) were used. Insured patients (45–85 years) who were shipped an mt-sDNA test during the data coverage period and had previously completed mt-sDNA screening with a negative result ≥ 2.5 years prior were included. Mt-sDNA re-screening adherence rate and mean time to test return were compared across payer types, and their associations with patient characteristics were assessed using multivariable regression models.

**Results:**

Of 793,567 patients (50–75 years: 89.0%; female: 62.0%), the re-screening adherence rate was 84.0% (from 66.5% for Medicaid to 90.2% for Medicare); mean (standard deviation) time to test return was 20.7 (20.8) days (from 19.2 [19.7] for Medicare to 22.4 [22.2] for Medicaid). Characteristics associated with higher likelihood of re-screening adherence included older ages (odds ratio [OR] = 1.25 and 1.11 for 65–75 and 76–85 years, respectively, relative to 45–49 years), living in a ZIP code with higher median household income (OR = 1.80 for > $200,000 relative to < $50,000), full digital outreach (OR = 1.84 relative to no digital outreach), and ≥ 3rd rounds of screening (OR = 2.44 relative to 2nd round of screening).

**Conclusion:**

Adherence to CRC re-screening with mt-sDNA test was high across payer types, with sustained adherence in later rounds of screening. Strategies to improve re-screening rates in subgroups associated with lower re-screening adherence are warranted.

## Introduction

In the USA, colorectal cancer (CRC) screening is recommended for average-risk individuals aged 45–75 years, with selective screening recommended for those aged 76–85 years depending on the patient’s health status, screening history, and preference [1, 2]. Based on these criteria, an estimated 60 million of the US population are eligible for CRC screening [3]. While the overall CRC screening rate has increased from approximately 50% in 2005 to 70% in 2021 among those aged 50–75 years [4], the rate remains below the 80% target set by the National Colorectal Cancer Roundtable [5]. Furthermore, prior to the change in age-eligibility recommendations for CRC screening to include average-risk individuals aged 45–49 years in 2021 [1, 2], the overall screening rate accounting for this age group was estimated at 59%, suggesting a wide CRC screening gap among these younger individuals [6].

CRC screening modalities include endoscopy (e.g., colonoscopy and sigmoidoscopy) as well as less invasive stool-based tests such as fecal immunochemical test (FIT), high-sensitivity guaiac fecal occult blood test (gFOBT), and multi-target stool DNA (mt-sDNA) test (marketed as Cologuard®; Exact Sciences, Madison, WI) that can be conducted at home [7]. For individuals at average risk of CRC, the US Preventive Services Task Force (USPSTF) and the American Cancer Society (ACS) recommend different CRC screening intervals based on the screening modalities—every 10 years for colonoscopy, every 5 years for sigmoidoscopy, annually for FIT and gFOBT, and every 3 years for the mt-sDNA test [1, 2].

While adherence to first-time screening is important to facilitate the diagnosis of CRC in the early stages when the cancer is more likely to be treated [8], it is equally important to maintain adherence to re-screening to maximize screening effectiveness [9]. Strategies to improve adherence to re-screening may, in the long run, help increase the rates of CRC detection, which may in turn reduce CRC mortality in the population over time [10]. With the added convenience of being home-based, less invasive modalities such as FIT/gFOBT and mt-sDNA tests may have a positive impact on re-screening adherence. A randomized trial has suggested that being adherent to re-screening with FIT may achieve similar effectiveness in detecting CRC compared to one-time sigmoidoscopy [11]. However, adherence often declines in successive rounds of screening [12, 13]. In a systematic review of 27 studies assessing repeat FIT/gFOBT testing, the median re-screening rate was 82% after 1 round of screening and declined to 47% after 2 rounds and 39% after 3 or more rounds of screening [12].

To date, limited research is available on adherence to re-screening with mt-sDNA test. Understanding the patient characteristics that are associated with repeat utilization of the mt-sDNA test is important to help identify potential strategies for improving re-screening adherence. Hence, this study sought to assess adherence to re-screening with the mt-sDNA test among previous mt-sDNA test users in a large, nationally insured US population across different payer types. In addition, as the timely return of the mt-sDNA test kit could contribute to the early detection of cancer or precancerous lesions that may help improve prognosis [14, 15], time to returning the mt-sDNA test was also assessed.

## Methods

### Data source

Laboratory data from Exact Sciences Laboratories LLC (ESL; Madison, WI) covering the period of January 1, 2023, to December 31, 2023, were used. All data were de-identified and compliant with the Health Insurance Portability and Accountability Act (HIPAA), and this study was considered exempt research.

### Study design and sample selection

**Fig. 1:**
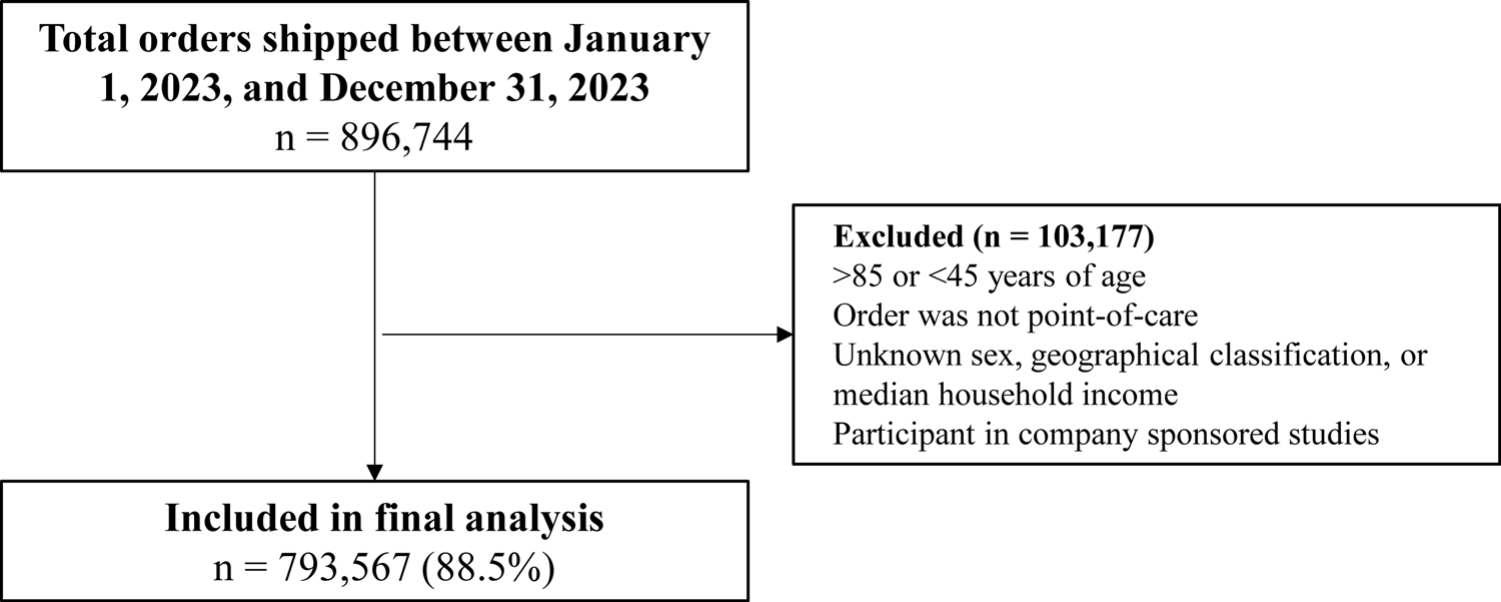
Sample selection

A retrospective cohort design was used. Patients were included in the study if they met the following criteria: (1) aged 45–85 years; (2) insured under a commercial payer, managed care organization, Medicare Advantage, Medicaid, or Medicare; (3) were shipped a Cologuard mt-sDNA test between January 1, 2023, and December 31, 2023; (4) had previously completed a valid mt-sDNA screening with a negative result ≥ 2.5 years prior; and (5) had no prior positive result across any Cologuard orders (Fig. 1).

Patients were excluded if (1) the mt-DNA test was not ordered from a point-of-care service (i.e., any order not placed as a result of an appointment with a health care provider); (2) there were missing data for sex, geography, or median household income; or (3) the patient was a participant in any of the prospective studies conducted by ESL (to minimize selection bias toward potentially more adherent patients).

### Measures and outcomes

Study measures and outcomes included patient demographic characteristics, mt-sDNA re-screening adherence rate, and time to test return. Patient demographic characteristics collected were age category, sex, race, ethnicity, preferred language, geography, patient median household income by ZIP code, outreach channels, and the number of rounds of screening (i.e., 2nd round or 3rd or more rounds).

Mt-sDNA re-screening adherence rate was defined as the percentage of eligible patients who completed and shipped the test kit upon re-screen, with the return test kit shipment received and a valid test result obtained by ESL within 180 days of the initial shipment date. Time to test return was defined as the number of days from the date of test kit shipment to the patient (i.e., the start date) to the date of receipt of a test kit that contained a specimen by ESL (i.e., the end date); the time to test return excluded the time for laboratory processing of the received tests.

### Statistical analyses

Study measures and outcomes were summarized using counts and percentages for categorical variables and means and standard deviations (SDs) for continuous variables.

Mt-sDNA re-screening adherence rate and mean number of days to test return were compared across different payer types for the following patient characteristics: age category, sex, race, ethnicity, preferred language, geography, patient median household income by ZIP code, outreach channels, and whether the current screening was the 2nd or the 3rd or more rounds of screening. Re-screening adherence rates were compared using the chi-square test or Fisher exact test (when observations were fewer than 5 in at least 1 cell of comparison table). Mean days to test return were compared using independent *t*-test (2 groups) or analysis of variance (≥ 3 groups).

Logistic regression was used to assess the association of patient characteristics with mt-sDNA re-screening adherence (binary outcome), and linear regression was used to assess the association with test return in days (continuous outcome). The output from linear regression was log-transformed because the time to screening was not normally distributed. For both regression models, covariates were included in the multivariable model if they had significant *p*-values in the descriptive, univariable analysis (race, ethnicity, and preferred language were excluded due to missing or incomplete data for most patients; payer type was not included as it was highly correlated with patient age). Final regression results were obtained from multivariable regression models run with all the following covariates: age category (reference [ref]: 45–49), sex (ref: female), geography (ref: metropolitan), patient median household income by ZIP code (ref: < $50,000), outreach channels (ref: no digital), and number of rounds of screening (ref: 2nd round).

All statistical analyses were conducted using R version 4.2.2 (The R Foundation for Statistical Computing, Vienna, Austria).

## Results

### Patient characteristics

The final sample included 793,567 patients who met the eligibility criteria, including 344,732 (43.4%) patients covered by commercial insurance, 19,819 (2.5%) by managed care organization, 230,276 (29.0%) by Medicare Advantage, 2745 (0.3%) by Medicaid, and 195,995 (24.7%) by Medicare (Table 1).

**Table 1:** Patient characteristics

	Overall	Commercial	Managed care organization	Medicare Advantage	Medicaid	Medicare
	*N* = 793,567	*N* = 344,732	*N* = 19,819	*N* = 230,276	*N* = 2745	*N* = 195,995
Age category, *n* (%)						
45–49 years	2711 (0.3)	2590 (0.8)	64 (0.3)	25 (0.0)	13 (0.5)	19 (0.0)
50–64 years	344,862 (43.5)	287,142 (83.3)	18,613 (93.9)	24,423 (10.6)	2299 (83.8)	12,385 (6.3)
65–75 years	360,895 (45.5)	49,113 (14.2)	1022 (5.2)	166,618 (72.4)	394 (14.4)	143,748 (73.3)
76–85 years	85,099 (10.7)	5887 (1.7)	120 (0.6)	39,210 (17.0)	39 (1.4)	39,843 (20.3)
Sex, *n* (%)						
Female	492,368 (62.0)	207,539 (60.2)	12,604 (63.6)	145,333 (63.1)	1762 (64.2)	125,130 (63.8)
Male	301,199 (38.0)	137,193 (39.8)	7215 (36.4)	84,943 (36.9)	983 (35.8)	70,865 (36.2)
Race, *n* (%)						
White	293,221 (36.9)	127,912 (37.1)	6660 (33.6)	81,480 (35.4)	1019 (37.1)	76,150 (38.9)
Black or African American	22,032 (2.8)	9352 (2.7)	1023 (5.2)	8412 (3.7)	183 (6.7)	3062 (1.6)
American Indian or Alaska Native	887 (0.1)	448 (0.1)	30 (0.2)	226 (0.1)	16 (0.6)	167 (0.1)
Asian Indian	8451 (1.1)	4608 (1.3)	361 (1.8)	1987 (0.9)	55 (2.0)	1440 (0.7)
Other	682 (0.1)	367 (0.1)	28 (0.1)	165 (0.1)	3 (0.1)	119 (0.1)
Unknown	468,294 (59.0)	202,045 (58.6)	11,717 (59.1)	138,006 (59.9)	1469 (53.5)	115,057 (58.7)
Ethnicity, *n* (%)						
Not Hispanic or Latino origin or descent	308,041 (38.8)	133,945 (38.9)	7725 (39.0)	86,983 (37.8)	1112 (40.5)	78,276 (39.9)
Hispanic or Latino origin or descent	20,410 (2.6)	10,198 (3.0)	811 (4.1)	6025 (2.6)	90 (3.3)	3286 (1.7)
Other	160 (0.0)	70 (0.0)	12 (0.1)	58 (0.0)	2 (0.1)	18 (0.0)
Unknown	464,956 (58.6)	200,519 (58.2)	11,271 (56.9)	137,210 (59.6)	1541 (56.1)	114,415 (58.4)
Preferred language, *n* (%)						
English	224,749 (28.3)	96,845 (28.1)	5874 (29.6)	65,485 (28.4)	778 (28.3)	55,767 (28.5)
Spanish	9477 (1.2)	4891 (1.4)	631 (3.2)	3093 (1.3)	58 (2.1)	804 (0.4)
Other	2696 (0.3)	917 (0.3)	413 (2.1)	753 (0.3)	53 (1.9)	560 (0.3)
Unknown	556,645 (70.1)	242,079 (70.2)	12,901 (65.1)	160,945 (69.9)	1856 (67.6)	138,864 (70.9)
Geography, *n* (%)						
Metropolitan	634,843 (80.0)	283,961 (82.4)	14,930 (75.3)	183,900 (79.9)	1976 (72.0)	150,076 (76.6)
Micropolitan	88,609 (11.2)	34,254 (9.9)	2558 (12.9)	26,482 (11.5)	371 (13.5)	24,944 (12.7)
Rural	28,103 (3.5)	10,464 (3.0)	926 (4.7)	7809 (3.4)	162 (5.9)	8742 (4.5%)
Small town	42,012 (5.3)	16,053 (4.7)	1405 (7.1)	12,085 (5.2)	236 (8.6)	12,233 (6.2)
Median household income by patient ZIP code, *n* (%)						
< $50,000	76,601 (9.7)	26,389 (7.7)	3580 (18.1)	29,620 (12.9)	519 (18.9)	16,493 (8.4)
$50,000–$75,000	325,384 (41.0)	132,618 (38.5)	9424 (47.6)	103,578 (45.0)	1213 (44.2)	78,551 (40.1)
$75,000–$100,000	215,033 (27.1)	97,798 (28.4)	4310 (21.7)	59,631 (25.9)	621 (22.6)	52,673 (26.9)
$100,000–$200,000	171,232 (21.6)	85,061 (24.7)	2463 (12.4)	36,855 (16.0)	385 (14.0)	46,468 (23.7)
> $200,000	5317 (0.7)	2866 (0.8)	42 (0.2)	592 (0.3)	7 (0.3)	1810 (0.9)
Outreach channels						
No digital	86,385 (10.9)	24,718 (7.2)	2197 (11.1)	31,522 (13.7)	365 (13.3)	27,583 (14.1)
Digital email only	55,804 (7.0)	19,061 (5.5)	1065 (5.4)	18,129 (7.9)	180 (6.6)	17,369 (8.9)
Digital SMS only	295,149 (37.2)	129,210 (37.5)	8836 (44.6)	88,100 (38.3)	1149 (41.9)	67,854 (34.6)
Digital SMS + email	356,229 (44.9)	171,743 (49.8)	7721 (39.0)	92,525 (40.2)	1051 (38.3)	83,189 (42.4)
Number of rounds of screening, *n* (%)						
2nd round	732,978 (92.4)	329,593 (95.6)	19,227 (97.0)	208,222 (90.4)	2672 (97.3)	173,264 (88.4)
3rd or more rounds	60,589 (7.6)	15,139 (4.4)	592 (3.0)	22,054 (9.6)	73 (2.7)	22,731 (11.6)

Table 1 summarizes the patient characteristics overall and by payer type. In the overall sample, 89% of patients were aged 50–75 years, with those covered by commercial insurance, managed care organization, and Medicaid being generally younger than those covered by Medicare and Medicare Advantage. Across payer types, approximately 60% were female. There was a high proportion of missingness in the race, ethnicity, and preferred language categories. The majority of patients lived in metropolitan areas (80% overall, > 70% across payer types) and a ZIP code with a median household income between $50,000 and $100,000 (68.1% overall, > 60% across payer types). Approximately 82% of patients received outreach via digital channels with SMS only or with both SMS and email. The current re-screening was the 2nd round of screening for the vast majority of patients.

### Re-screening adherence rate and mean time to test return

Overall, the mt-sDNA re-screening adherence rate was 84.0% (Table 2), and the rate was similar in patients excluded from the analysis due to missing data (*N* = 4149; 80.5%). Re-screening adherence rates varied across payer type with the highest for Medicare (90.2%) and the lowest for Medicaid (66.5%) (*p* < 0.001). Re-screening adherence rate was above 80% for all age groups in the overall sample, with those aged 65–75 years having the highest adherence numerically (85.1%), although the rates in each age group varied by payer type. Females and males appeared to have similar adherence rates overall (83.8% vs 84.2%); this observation was also consistent across payer types. Similar adherence rates were also observed for patients living in different geographical locations (i.e., metropolitan, micropolitan, rural areas, and small towns) overall and within each payer type. Patients living in areas with higher median household income had numerically higher adherence, with rates of 87.7% overall and 84.8–92.7% across payer types in the > $200,000 category, relative to 80.0% overall and 58.6–86.5% across payer types in the < $50,000 category. With respect to the type of outreach, patients receiving full digital outreach with both SMS and email had the numerically highest re-screening adherence rates (86.3% overall and 73.4–92.8% across payer types) relative to those with partial or no digital outreach. Those completing the 3rd or more rounds of screening (i.e., the 3rd or more overall lifetime mt-sDNA test) had an adherence rate of 92.6% overall and 71.2–95.8% across payer types, whereas those completing the 2nd round of screening had an adherence rate of 83.2% overall and 66.4–89.5% across payer types.

**Table 2:** Re-screening adherence rates overall and by payer type

	Overall	Commercial	Managed care organization	Medicare Advantage	Medicaid	Medicare	
	*N* = 793,567	*N* = 344,732	*N* = 19,819	*N* = 230,276	*N* = 2745	*N* = 195,995	*p*-value
Overall, %	84.00	81.00	76.60	83.90	66.50	90.20	< 0.001
Demographic characteristics							
Age category, %							
45–49 years	82.90	83.20	79.70	64.00	53.80	89.50	*0.007*
50–64 years	82.80	84.20	77.50	75.60	70.70	75.40	< *0.001*
65–75 years	85.10	67.90	60.70	85.00	44.90	91.50	< *0.001*
76–85 years	83.70	36.50	60.80	84.10	41.00	90.40	< *0.001*
Sex, %							
Female	83.80	80.60	77.00	83.70	67.60	90.20	< *0.001*
Male	84.20	81.60	75.90	84.20	64.50	90.20	< *0.001*
Race, %							
White	83.90	80.50	75.70	83.60	69.20	90.70	< *0.001*
Black or African American	78.70	79.00	74.00	78.00	57.40	82.80	< *0.001*
American Indian or Alaska Native	74.70	76.10	73.30	74.30	56.20	73.70	*0.477*
Asian Indian	84.10	84.00	76.20	84.10	67.30	87.00	< *0.001*
Other	81.70	79.60	82.10	81.20	66.70	89.10	*0.132*
Unknown	84.30	81.40	77.30	84.40	65.80	90.20	< *0.001*
Ethnicity, %							
Not Hispanic or Latino origin or descent	84.70	81.60	76.80	84.80	67.40	91.10	< *0.001*
Hispanic or Latino origin or descent	80.40	79.20	76.70	79.60	71.10	86.40	< *0.001*
Other	86.20	87.10	75.00	87.90	100.00	83.30	*0.700*
Unknown	83.60	80.80	76.40	83.50	65.50	89.80	< *0.001*
Preferred language, %							
English	84.30	80.80	76.30	84.20	71.50	91.60	< *0.001*
Spanish	77.90	77.30	79.90	78.70	72.40	77.10	*0.295*
Other	77.50	77.80	73.10	80.70	71.70	76.60	*0.034*
Unknown	84.00	81.20	76.70	83.90	64.10	89.80	< *0.001*
Geography, %							
Metropolitan	83.80	80.90	76.60	83.80	66.20	90.10	< *0.001*
Micropolitan	84.50	81.20	76.90	84.10	67.10	90.40	< *0.001*
Rural	85.40	82.10	76.10	84.90	68.50	91.10	< *0.001*
Small town	84.80	81.70	76.50	84.30	66.10	90.70	< *0.001*
Median household income by patient ZIP code, %							
< $50,000	80.00	77.60	72.10	79.80	58.60	86.50	< *0.001*
$50,000–$75,000	83.40	80.30	76.20	83.40	64.20	89.90	< *0.001*
$75,000–$100,000	84.80	81.70	78.50	85.20	70.40	90.80	< *0.001*
$100,000–$200,000	85.60	82.40	80.80	86.20	77.70	91.50	< *0.001*
> $200,000	87.70	84.80	85.70	87.00	85.70	92.70	< *0.001*
Outreach channels, %							
No digital	77.60	70.00	68.70	78.30	59.50	84.70	< *0.001*
Digital email only	82.30	75.40	73.40	82.50	59.40	90.30	< *0.001*
Digital SMS only	83.30	81.10	76.30	82.80	63.50	89.40	< *0.001*
Digital SMS + email	86.30	83.20	79.60	87.10	73.40	92.80	< *0.001*
Number of rounds of screening, %							
2nd round	83.20	80.70	76.10	83.00	66.40	89.50	< *0.001*
3rd or more rounds	92.60	88.40	90.70	92.30	71.20	95.80	< *0.001*

The mean (SD) time to test return from the shipment of the mt-sDNA kit to the receipt of the valid test was 20.7 (20.8) days overall (Table 3). Across payer types, the mean (SD) time to test return ranged from 19.2 (19.7) days for those covered by Medicare to 22.4 (22.2) days for those covered by Medicaid (*p* < 0.001). Patients receiving full digital outreach with both SMS and email relative to those receiving partial or no digital outreach, and patients completing 3rd or more rounds of screening relative to those completing 2nd rounds of screening, had numerically shorter time to test return, both overall and within each payer type.

**Table 3:** Time to test return overall and by payer type

	Overall	Commercial	Managed care organization	Medicare Advantage	Medicaid	Medicare	
	*N* = 793,567	*N* = 344,732	*N* = 19,819	*N* = 230,276	*N* = 2745	*N* = 195,995	*p*-value
Overall, mean (SD)	20.7 (20.8)	22.3 (21.8)	21.4 (21.8)	19.5 (20.1)	22.4 (22.2)	19.2 (19.7)	< 0.001
Demographic characteristics							
Age category, mean (SD)							
45–49 years	22.2 (20.9)	22.3 (21.0)	16.3 (9.9)	30.2 (29.0)	14.6 (5.8)	19.9 (16.5)	*0.107*
50–64 years	22.5 (22.2)	22.5 (22.0)	21.4 (21.9)	22.1 (23.0)	22.6 (21.8)	23.8 (25.3)	< *0.001*
65–75 years	19.6 (19.9)	20.9 (20.6)	20.2 (21.7)	19.6 (20.1)	20.9 (24.9)	19.2 (19.5)	< *0.001*
76–85 years	18.0 (18.1)	19.2 (20.1)	20.6 (22.6)	17.9 (17.8)	22.6 (29.8)	18.0 (18.3)	*0.005*
Sex, mean (SD)							
Female	21.2 (21.3)	22.8 (22.2)	21.6 (22.0)	20.1 (20.6)	22.2 (21.5)	19.8 (20.2)	< *0.001*
Male	19.8 (20.0)	21.5 (21.1)	20.9 (21.6)	18.5 (19.1)	22.9 (23.3)	18.2 (18.7)	< *0.001*
Race, mean (SD)							
White	20.8 (21.0)	22.5 (22.1)	22.0 (22.6)	19.5 (20.2)	22.0 (20.8)	19.2 (19.7)	< *0.001*
Black or African American	19.8 (20.0)	21.0 (20.7)	19.9 (20.6)	18.9 (19.4)	21.5 (23.7)	18.5 (18.9)	< *0.001*
American Indian or Alaska Native	22.7 (21.7)	21.5 (17.2)	22.9 (15.6)	23.3 (24.6)	16.3 (7.8)	25.5 (28.9)	*0.403*
Asian Indian	19.7 (20.1)	20.4 (20.0)	19.0 (21.9)	19.0 (20.2)	21.3 (31.3)	18.6 (19.4)	*0.025*
Other	20.5 (21.7)	18.2 (14.2)	19.4 (17.0)	22.0 (25.0)	11.5 (7.8)	25.5 (32.1)	*0.039*
Unknown	20.6 (20.8)	22.2 (21.7)	21.2 (21.5)	19.5 (20.0)	23.0 (22.6)	19.3 (19.7)	< *0.001*
Ethnicity, mean (SD)							
Not Hispanic or Latino origin or descent	20.4 (20.6)	22.1 (21.7)	21.5 (22.0)	19.2 (20.0)	21.7 (21.4)	19.0 (19.4)	< *0.001*
Hispanic or Latino origin or descent	21.2 (20.6)	22.1 (21.0)	21.9 (21.7)	20.3 (20.2)	21.6 (23.1)	20.0 (19.4)	< *0.001*
Other	22.5 (23.1)	28.6 (29.5)	17.7 (5.5)	16.1 (10.2)	60.5 (68.6)	16.9 (14.3)	*0.004*
Unknown	20.8 (21.0)	22.4 (22.0)	21.3 (21.8)	19.7 (20.2)	23.0 (22.5)	19.4 (19.9)	< *0.001*
Preferred language, mean (SD)							
English	20.4 (20.6)	22.0 (21.7)	21.3 (21.7)	19.3 (19.9)	22.6 (22.5)	18.9 (19.2)	< *0.001*
Spanish	21.8 (21.3)	22.0 (20.4)	21.3 (20.0)	21.3 (22.0)	24.6 (27.5)	22.0 (24.1)	*0.560*
Other	18.7 (19.3)	18.6 (18.2)	18.2 (20.4)	18.0 (18.8)	27.1 (33.2)	19.7 (19.3)	*0.050*
Unknown	20.8 (20.9)	22.4 (21.9)	21.5 (22.0)	19.6 (20.1)	22.1 (21.4)	19.3 (19.8)	< *0.001*
Geography, mean (SD)							
Metropolitan	20.7 (21.0)	22.3 (22.0)	21.2 (21.9)	19.5 (20.2)	22.2 (22.6)	19.3 (20.0)	< *0.001*
Micropolitan	20.2 (20.1)	21.9 (20.8)	21.5 (21.3)	19.3 (20.0)	22.0 (18.9)	19.0 (19.2)	< *0.001*
Rural	20.8 (19.4)	22.7 (21.0)	21.9 (20.4)	19.8 (18.8)	23.3 (23.0)	19.5 (17.6)	< *0.001*
Small town	20.3 (19.8)	21.7 (20.2)	22.1 (22.8)	19.5 (20.1)	24.6 (23.1)	19.0 (18.6)	< *0.001*
Median household income by patient ZIP code, mean (SD)							
< $50,000	20.5 (20.3)	21.7 (20.6)	21.0 (21.5)	20.0 (20.4)	21.7 (22.2)	19.4 (19.5)	< *0.001*
$50,000–$75,000	20.5 (20.5)	22.2 (21.5)	21.5 (22.0)	19.4 (20.0)	23.1 (21.3)	19.0 (19.3)	< *0.001*
$75,000–$100,000	20.8 (21.0)	22.4 (22.0)	21.2 (21.4)	19.6 (20.2)	21.5 (23.6)	19.3 (19.7)	< *0.001*
$100,000–$200,000	20.9 (21.3)	22.5 (22.3)	21.6 (22.4)	19.4 (20.0)	23.1 (22.5)	19.4 (20.3)	< *0.001*
> $200,000	20.8 (21.4)	22.1 (22.4)	21.1 (15.2)	18.7 (18.8)	11.0 (1.4)	19.4 (20.8)	< *0.001*
Outreach channels, mean (SD)							
No digital	22.0 (23.7)	25.5 (26.4)	22.0 (24.2)	20.5 (22.5)	27.3 (28.1)	20.7 (22.3)	< *0.001*
Digital email only	21.1 (22.6)	23.9 (24.9)	20.9 (21.3)	19.5 (20.9)	23.5 (23.1)	20.0 (21.8)	< *0.001*
Digital SMS only	20.7 (20.5)	22.1 (21.3)	21.5 (21.3)	19.8 (20.0)	22.0 (21.2)	19.2 (19.1)	< *0.001*
Digital SMS + email	20.3 (20.1)	21.8 (21.1)	21.1 (21.9)	19.0 (19.2)	21.3 (20.9)	18.7 (18.7)	< *0.001*
Number of rounds of screening, mean (SD)							
2nd round	21.0 (21.1)	22.5 (22.0)	21.5 (21.9)	19.9 (20.4)	22.6 (22.3)	19.6 (20.0)	< *0.001*
3rd or more rounds	16.8 (16.8)	18.4 (17.7)	17.4 (18.5)	16.3 (16.4)	18.7 (17.6)	16.4 (16.5)	< *0.001*

Abbreviation: *SD* standard deviation

### Summary of model output

The associations of patient characteristics with re-screening adherence and time to test return are shown in Table 4. Compared with patients aged 45–49 years, those aged 65–75 years were 25% more likely to be adherent to mt-sDNA re-screening (odds ratio [OR]: 1.25, 95% confidence interval [CI]: 1.13–1.38, *p* < 0.001), and those aged 76–85 years were 11% more likely to be adherent (OR: 1.11, 95% CI: 1.01–1.23, *p* = 0.04). Patients living in metropolitan areas were least likely to be adherent to re-screening (the likelihood of adherence in these patients was 18%, 32%, and 26% lower than those living in micropolitan areas, rural areas, and small towns, respectively; all *p* < 0.001). The likelihood to be adherent to mt-sDNA re-screening increased with increasing median household income by patient ZIP code, ranging from 24% higher likelihood in those living in areas with a median household income of $50,000–$75,000 (OR: 1.24, 95% CI: 1.22–1.27, *p* < 0.001) to 80% higher likelihood in those living in areas with a median household income of > $200,000 (OR: 1.80, 95% CI: 1.66–1.97, *p* < 0.001), compared with those living in areas with median household income < $50,000. Patients receiving both SMS and email outreach had an 84% higher likelihood of adherence compared with those receiving no digital outreach (OR: 1.84, 95% CI: 1.80–1.87, *p* < 0.001). Those at 3rd or more rounds of screening had almost a 2.4-fold increase in their likelihood of adherence than those at the 2nd round of screening (OR: 2.44, 95% CI: 2.36–2.51, *p* < 0.001).

**Table 4:** Association of patient characteristics with re-screening adherence and time to test return

	Re-screening adherence rate			Log-transformed days to test return		
	Logistic regression			Linear regression		
	Odds ratio	95% CI	*p*-value	exp(estimate)	95% CI	*p*-value
Age category						
45–49 years	Ref	-	-	Ref	-	-
50–64 years	1.04	(0.94, 1.15)	0.474	1.00	(0.97, 1.03)	0.542
65–75 years	1.25	(1.13, 1.38)	< 0.001	0.88	(0.85, 0.91)	< 0.001
76–85 years	1.11	(1.01, 1.23)	0.039	0.83	(0.80, 0.85)	< 0.001
Sex						
Female	Ref	-	-	Ref	-	-
Male	1.05	(1.03, 1.06)	< 0.001	0.93	(0.93, 0.94)	< 0.001
Geography						
Metropolitan	Ref	-	-	Ref	-	-
Micropolitan	1.18	(1.15, 1.20)	< 0.001	1.00	(0.99, 1.00)	0.991
Rural	1.32	(1.28, 1.37)	< 0.001	1.05	(1.04, 1.06)	< 0.001
Small town	1.26	(1.22, 1.29)	< 0.001	1.01	(1.00, 1.02)	0.008
Median household income by patient ZIP code						
< $50,000	Ref	-	-	Ref	-	-
$50,000–$75,000	1.24	(1.22, 1.27)	< 0.001	1.00	(0.99, 1.00)	0.315
$75,000–$100,000	1.40	(1.38, 1.48)	< 0.001	1.02	(1.01, 1.02)	< 0.001
$100,000–$200,000	1.51	(1.48, 1.55)	< 0.001	1.01	(1.00, 1.02)	< 0.001
> $200,000	1.80	(1.66, 1.97)	< 0.001	0.99	(0.97, 1.01)	0.478
Outreach channels						
No digital	Ref	-	-	Ref	-	-
Digital email only	1.30	(1.26, 1.33)	< 0.001	0.97	(0.96, 0.98)	< 0.001
Digital SMS only	1.49	(1.46, 1.51)	< 0.001	0.98	(0.97, 0.99)	< 0.001
Digital SMS + email	1.84	(1.80, 1.87)	< 0.001	0.96	(0.96, 0.97)	< 0.001
Number of rounds of screening						
2nd round	Ref	-	-	Ref	-	-
3rd or more rounds	2.44	(2.36, 2.51)	< 0.001	0.86	(0.85, 0.86)	< 0.001

Abbreviations: *CI* confidence interval, *Ref* reference

Compared with patients aged 45–49 years, the mean time to test return was 12% lower (i.e., shorter) in those aged 65–75 years (95% CI: 0.85–0.91, *p* < 0.001) and 17% lower in those aged 76–85 years (95% CI: 0.80–0.85, *p* < 0.001). Individuals who received some form of digital outreach took a shorter time to return the test when compared to those who received no digital outreach, with those who received both SMS and email taking the shortest amount of time (4% shorter, 95% CI: 0.96–0.97, *p* < 0.001). Time to test return was also significantly shorter among males relative to females (7% shorter, 95% CI: 0.93–0.94, *p* < 0.001), and those at their 3rd or more rounds of screening relative to those at their 2nd round of screening (14% shorter, 95% CI: 0.85–0.86, *p* < 0.001). Patients living in rural areas and small towns had 5% (95% CI: 1.04–1.06, *p* < 0.001) and 1% (95% CI: 1.00–1.02, *p* = 0.008) longer times to test return compared to those living in metropolitan areas, and those living in ZIP code with median household income of $75,000–$100,000 and $100,000–$200,000 had 2% (95% CI: 1.01–1.02, *p* < 0.001) and 1% (95% CI: 1.00–1.02, *p* < 0.001) longer time to test return than those living in ZIP code with median household income of $50,000.

## Discussion

In this large US retrospective cohort study encompassing patients at age ranges recommended for CRC screening across all major payer types, 84% of patients who had previously completed an mt-sDNA test were adherent to re-screening, and the time to test return was 21 days on average. Re-screening adherence was high across all payer types, with patients covered by Medicare having the highest re-screening adherence rate of 90%. Across all payer types, re-screening adherence rates were generally higher with increasing household income, full digital outreach, and more rounds of screening. Digital outreach with both SMS and email increased the likelihood of re-screening adherence by almost twofold compared with no digital outreach. Patients at their 3rd or more round of screening (i.e., the 3rd or more overall lifetime mt-sDNA test) were more than twice as likely to be adherent to re-screening than those at their 2nd round of screening. The mean time to test return was shorter among patients receiving both SMS and email outreach relative to no digital outreach and those who were at later rounds of screening beyond the 2nd round. Nonetheless, the overall time to test return was largely below a month across patients. Together, these results suggest a high overall adherence to re-screening with the mt-sDNA test and also provide important insight into patient characteristics associated with repeat mt-sDNA use, which may help inform strategies to improve re-screen rates and early CRC detection. Although the current data did not allow for the assessment of follow-up colonoscopy, a delay in time to colonoscopy beyond 9 months following a positive screening test has been associated with a more advanced disease stage at diagnosis [16, 17]; thus, timely diagnostic follow-up among patients with a positive test result is necessary to facilitate CRC detection.

The re-screening adherence rate with point-of-care–ordered mt-sDNA test in this study was consistent with that reported for FIT/gFOBT in a systemic review, which found a median adherence of 82% for the 2nd round of FIT/gFOBT screening [12]. However, our study found that adherence to the 3rd or more rounds of screening with mt-sDNA was 93%, which was considerably higher than the 39% reported for the same number of rounds of screening with FIT/gFOBT. It should be noted that the different study designs and populations (e.g., we included data from ESL laboratory records), as well as unmeasured confounders in this study (e.g., patients’ fitness level and CRC history), may have influenced the adherence rates observed. Furthermore, some non-US studies have found that FIT/gFOBT screening history in organized population-based programs predicted participation in subsequent rounds of re-screening, with higher participation among those with more prior rounds of screening [18, 19], which aligns with the observations in the current study. The high re-screening adherence rate found in this study among routine care patients is encouraging, given existing evidence suggests that cross-sectional adherence may also be higher with mt-sDNA test (51–67% [20, 21]) than with FIT/gFOBT (12–41% [22–26]). Collectively, the high repeat and cross-sectional adherence to mt-sDNA testing could be crucial to improve the overall CRC re-screening rate in the population.

Across patient characteristics, patients covered by Medicaid showed lower mt-sDNA re-screening adherence than those covered by Medicare, Medicare Advantage, commercial insurance, and managed care organizations. This observation is consistent with prior studies demonstrating lower screening adherence with public insurance, particularly Medicaid, relative to private insurance [27–29]. Aligning with the literature, our results have identified Medicaid patients as a population that may benefit the most from interventions aiming to improve uptake and adherence to CRC screening.

The large sample size of our study has allowed us to identify several patient subgroups associated with lower re-screening adherence that may warrant more customized strategies to improve re-screening rates. For instance, younger patients (i.e., aged < 65 years) were found to have a lower likelihood of re-screening adherence and longer time to test return than older patients, who may be more often encouraged by health care providers to screen because of the increased CRC risk with advancing age [30]. In addition, the ACS guidelines in 2018 and the USPSTF in 2021 lowered the age-eligibility recommendations for CRC screening to 45 years of age [1, 2]. The lower re-screening adherence we found in the younger age group may in part be due to a lack of awareness in patients, and even healthcare providers, of the relatively recent guideline updates. Hence, efforts should be made to increase patient and clinician education regarding the importance of re-screening in younger individuals. Meanwhile, patients living in metropolitan areas were less likely to be adherent to re-screening than those living in other areas. More research is needed to understand this observation, which may help formulate potential strategies to encourage CRC re-screening among metropolitan patients. Additionally, we also observed lower re-screening adherence in patients living in ZIP code with lower median household income. Lower socioeconomic status is a well-documented barrier to health care access, and out-of-pocket costs could be a factor deterring patients with low income from adhering to CRC re-screening [31]. In this regard, insurers should weigh the cost-benefit of complete reimbursement for screening over the potential costs of delayed CRC detection.

To the best of our knowledge, this study was among the first to report CRC re-screening adherence to the mt-sDNA test kit in a real-world setting. Adequate adherence to re-screening is essential to maximize screening effectiveness by identifying cancers or precancerous lesions manifesting over time and doing so in the early stages when they are easier and potentially less costly to manage [10, 14, 15, 32]. The generally high re-screening adherence with the mt-sDNA test found in this study sheds light on the potential of the test in mitigating the clinical and economic burden associated with CRC management. Future research should identify interventions to further increase CRC re-screening rates overall, especially in patient groups with low adherence rates, such as Medicaid and lower-income populations.

### Limitations

The findings of this study should be interpreted with limitations. As our study sample included patients who had previously completed an mt-sDNA test, it may represent a population that had a high likelihood to adhere to CRC screening. In addition, this study used laboratory data from ESL, who is the manufacturer of the mt-sDNA test; therefore, no other screening modalities were assessed during the same timeframe. There was also high missingness for race, ethnicity, and preferred language information in the data, and thus the impact of these factors on study outcomes could not be evaluated.

## Conclusions

This large retrospective study showed high adherence to CRC re-screening with the mt-sDNA test kit and sustained adherence in later rounds of screening. Re-screening adherence rates were high across all payer types, with the highest rate among those covered by Medicare. The study also identified important patient subgroups associated with lower re-screening adherence, including patients covered by Medicaid, younger patients, and those with lower income, who may benefit from further customized outreach strategies to improve re-screening rates.

## Data Availability

The data that support the findings of this study are available from Exact Sciences Laboratories LLC. Restrictions apply to the availability of these data, which were used under license for this study. Data are available from the authors with the permission of Exact Sciences Laboratories LLC.
